# Potential Therapeutic Targets of Epigallocatechin Gallate (EGCG), the Most Abundant Catechin in Green Tea, and Its Role in the Therapy of Various Types of Cancer

**DOI:** 10.3390/molecules25143146

**Published:** 2020-07-09

**Authors:** Saleh A. Almatroodi, Ahmad Almatroudi, Amjad Ali Khan, Fahad A. Alhumaydhi, Mohammed A. Alsahli, Arshad Husain Rahmani

**Affiliations:** 1 Department of Medical Laboratories, College of Applied Medical Sciences, Qassim University, Buraydah 52571, Saudi Arabia; smtrody@qu.edu.sa (S.A.A.); aamtrody@qu.edu.sa (A.A.); f.alhumaydhi@qu.edu.sa (F.A.A.); shly@qu.edu.sa (M.A.A.); 2 Department of Basic Health Science, College of Applied Medical Sciences, Qassim University, Buraydah 52571, Saudi Arabia; akhan@qu.edu.sa

**Keywords:** EGCG, cancer, cell signaling pathways, bioavailability, synergistic effect, clinical trials

## Abstract

Epigallocatechin-3-gallate (EGCG), an active compound of green tea and its role in diseases cure and prevention has been proven. Its role in diseases management can be attributed to its antioxidant and anti-inflammatory properties. The anti-cancer role of this green tea compound has been confirmed in various types of cancer and is still being under explored. EGCG has been proven to possess a chemopreventive effect through inhibition of carcinogenesis process such as initiation, promotion, and progression. In addition, this catechin has proven its role in cancer management through modulating various cell signaling pathways such as regulating proliferation, apoptosis, angiogenesis and killing of various types of cancer cells. The additive or synergistic effect of epigallocatechin with chemopreventive agents has been verified as it reduces the toxicities and enhances the anti-cancerous effects. Despite its effectiveness and safety, the implications of EGCG in cancer prevention is certainly still discussed due to a poor bioavailability. Several studies have shown the ability to overcome poor bioavailability through nanotechnology-based strategies such as encapsulation, liposome, micelles, nanoparticles and various other formulation. In this review, we encapsulate therapeutic implication of EGCG in cancer management and the mechanisms of action are discussed with an emphasis on human clinical trials.

## 1. Introduction

Cancer is a leading public health problem worldwide in term of morbidity and mortality. Many factors are involved in this pathogenesis, including alterations in cell signaling pathways and other various biological process. It is a notorious killer disease, and various risk factors linked with cancer seem to be increasing day by day. The mortality rate due to this menace is increasing rapidly and is estimated at 9.55 million deaths every year [1]. The exact reasons behind the formation of cancer are still not clear but it is thought that carcinogens are] a major culprit in this notorious disease.

Chemotherapeutic drugs such as doxorubicin, cisplatin, 5-fluorouracil, and paclitaxel, and radiotherapy are commonly used modes of treatment in most of the cancers. The chemotherapeutic drugs cause adverse effects, are cytotoxic even for the normal cells, change the physiological, biochemical processes, and alter the cell signaling pathways. However, the search for safe anti-cancer drugs at an affordable price is still a prime interest in cancer treatment. To overcome the toxicity, side effects and cost of existing anti-cancer drugs, natural compounds are a good choice in cancer management. They may regulate numerous key cell-signaling molecules with few side effects.

**Figure 1:**
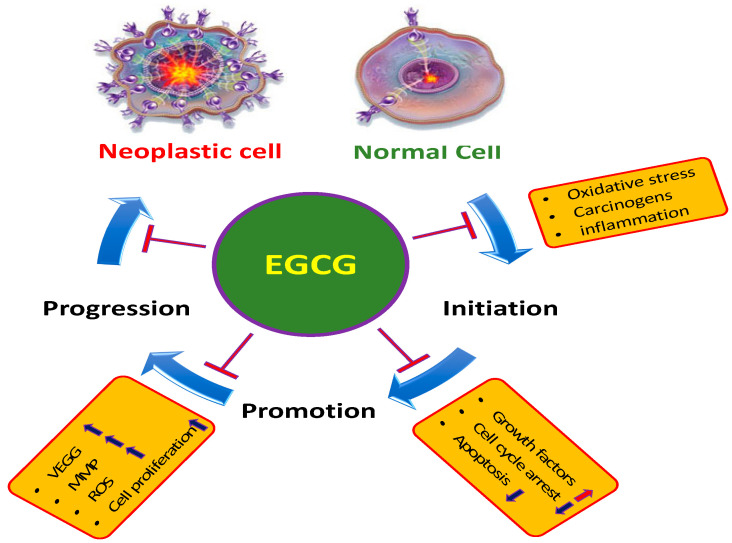
Epigallocatechin-3-gallate (EGCG)’s role in inhibition of cancer formation through inhibiting carcinogenesis process.

Medicinal plants and their principal compounds have clearly confirmed to be helpful in managing the diseases and inhibiting the carcinogenesis process such as initiation, promotion and progression (Figure 1). Moreover, natural products or active compound of herbs show a substantial starring role in the enhancement of efficacy of anti-cancer drugs and also reduce the effect of toxicity. Drug combinations that comprise dietary supplements and natural products have been expected to achieve the same effects as conventional chemotherapeutic drugs with reduced adverse effects [2].

## 2. Main Mechanisms of EGCG in the Inhibition of Cancer

### 2.1. Inflammation

**Figure 2:**
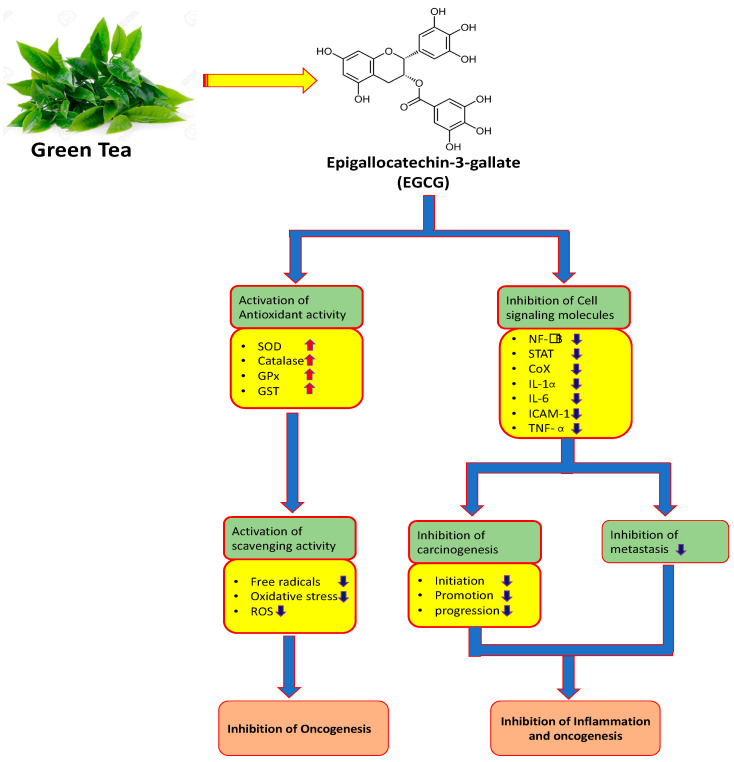
EGCG shows role in the inhibition of inflammation and inhibition of oncogenesis through the abrogation of the reactive oxygen.

Inflammation a vital hallmark of progression and development of cancer, and raised inflammatory mediators are related to bad prognosis in patients with cancer [3]. Some of the cancer related inflammatory factors involved in the development of cancer are tumor necrosis factor, chemokines, inflammasomes, transcription factors, cytokines, infiltrating or circulating immune cells, and ROS [4] (Figure 2).

Tumor necrosis factor (TNF-α) is an important pro-inflammatory cytokine and is associated with inflammatory diseases or its altered function has been noticed in various cancers. However, suppression of NF-κb is an imperative footstep in the inhibition of cancer development and progression. Therefore, discovery of new anti-inflammatory compounds might be an auspicious line to inhibit the of inflammation-related cancers. EGCG, the essential compound of green tea has been found to possess anti-inflammatory potentiality and inhibits the pro-inflammatory cytokine activity. A study provided evidence that EGCG plays a starring role in the inhibition of TNF-α and can protect TNF-α-mediated lung inflammation through down-regulation of oxidative stress and intercellular adhesion molecule-1 expression (ICAM-1) [5] (Table 1). EGCG has been found to inhibit tumor necrosis factor-α (TNF-α)-induced production of monocyte chemoattractant protein-1 (MCP-1) in human umbilical vein endothelial cells. The result confirmed that EGCG meaningfully decreased the TNF-α-induced protein and mRNA expression of MCP-1. Moreover, EGCG suppresses TNF-α-induced MCP-1 expression in human umbilical vein endothelial cells and such effect was arbitrated by 67LR and was via the inhibition of NF-κB activation [6]. Cyclooxygenase (COX)-2 overexpression has been noted in various cancers. However, its regulation is an important step towards cancer management. EGCG inhibits cyclooxygenase-2 without affecting COX-1 expression at both the mRNA and protein levels, in androgen-sensitive LNCaP and androgen-insensitive PC-3 human prostate carcinoma cells. Based on this finding, it is appealing to propose that a combination of EGCG with chemotherapeutic drugs could be a better plan for prevention and treatment of prostate cancer [7].

### 2.2. Reactive Oxygen Species (ROS) 

Reactive oxygen species are principally produced as by-products, have numerous harmful effects, and lead to the pathogenesis of various types of diseases [8] (Figure 2). The ROS production/antioxidant defense system balance is required for homeostasis [9]. However, neutralization or removal of ROS is an important step towards inhibition of pathogenesis. The antioxidant enzymes play a noteworthy starring role in the neutralization of ROS or counteract its generation. However, the antioxidant properties of natural products show a dynamic role in the suppression of oxidative stress and the ability to inhibit the pathogenesis. 

EGCG is a well-known antioxidant and it scavenges most free radicals, such as ROS and RNS. EGCG is capable of restoring the enzymatic activity of glutathione peroxidase and can regulate the glutathione levels. In addition, EGCG plays a role in the inhibition of H_2_O_2_ and NO production in human skin [10]. Based on an experiment of electron spin resonance (ESR) spin-trapping, to investigate the scavenging activity of EGCG for hydroxyl and superoxide radicals, it efficiently scavenges these radicals with the help of the xanthine oxidase system. Through its antioxidant properties, EGCG exhibited a protective effect against DNA damage [11]. The apoptotic regulation by EGCG on colon cancer cells in the presence of low-dose H_2_O_2_ was investigated and it was observed to induced apoptosis and abolished the cell-proliferative effect [12]. EGCG and the anticancer drug tamoxifen were both tested as potential chemopreventive agents. The doses required for a fifty percent decrease in hydrogen peroxide were EGCG with a dose of 25 µM, which eliminated TPA-mediated hydrogen peroxide production, and even lowered the normal cellular levels [13]. 

### 2.3. Angiogenesis

Cancer always needs a blood supply to grow and when it is deprived of angiogenesis, cancer can remain dormant. Inhibition of angiogenesis process is considered as strength in cancer treatment strategies. Angiogenesis shows a serious featuring role in the pathogenesis of numerous diseases.

To stop the angiogenesis, bevacizumab is commonly used to shut off the tumor’s blood supply but this drug causes severe side effects. Various types of natural compounds have been tried as inhibitors of altered angiogenesis in different pathological conditions including cancer. In this regard, EGCG shows a dynamic starring role in the inhibition or uncontrolled angiogenesis process and inhibits the pro-angiogenic factors of VEGF. Studies based on breast carcinoma cell lines reported that high levels of EGCG have been proven to reduce VEGF production [14] (Table 1). EGCG has been described to interfere with VEGFR-2 activity [15]. Moreover, EGCG inhibits the activation of HIF-1α and NFκB, and VEGF expression, thereby suppressing tumor angiogenesis and breast cancer progression. The result has further revealed that EGCG treatment meaningfully reduced tumor weight over the control and tumor VEGF expression [16]. EGCG inhibits VEGF production through decreasing both the constitutive activation of Stat3 and NF-kappa B, in cancer cells. Consequently, EGCG may be valuable in treating head and neck squamous cell carcinoma and breast carcinoma because it can cause both antiproliferative and antiangiogenic activities [17]. Epigallocatechin-3 gallate concentration-dependently inhibited vascular endothelial growth factor-induced DNA synthesis, cell proliferation, and autophosphorylation of vascular endothelial growth factor receptors-1 and -2 [18].

### 2.4. Apoptosis

**Figure 3:**
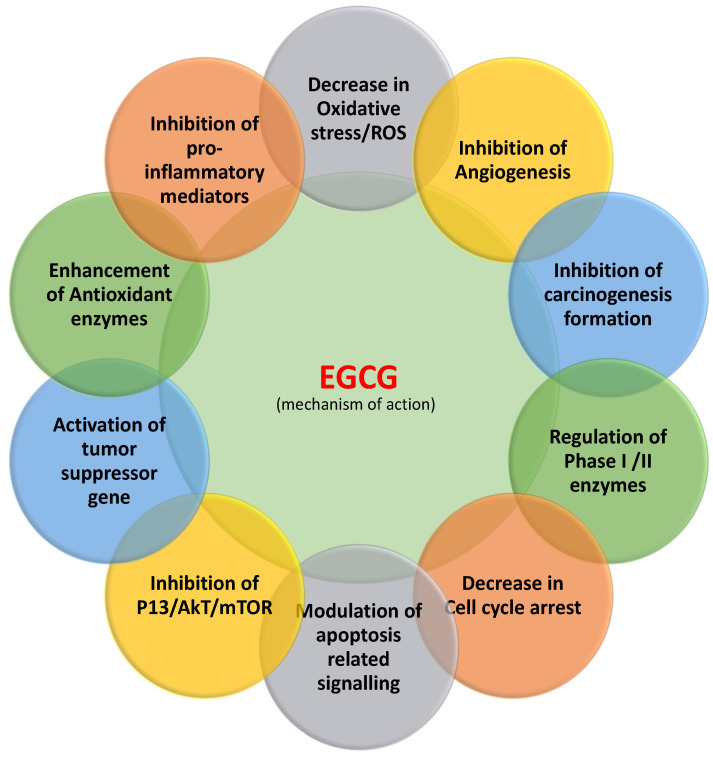
Mechanism of action of EGCG in cancer management through modulating various cell signaling pathways.

Apoptosis is known as controlled cell death and organized physiological process involve in the removal of damaged cells (Figure 3). The alteration in pro-apoptotic, anti-apoptotic proteins and, decreased caspase function has been noticed in many cancers. Natural/active compounds from medicinal plants play important role in cancer inhibition through induction of apoptosis. The induction of cancer cell apoptosis is the chief concern in anticancer compound research. A study based on the role of resveratrol in cancer described its anticancer action in pancreatic cancer cells through suppression of the expression of NAF-1 in pancreatic cancer cells via inducing cellular reactive oxygen species (ROS) accumulation and activating Nrf2 signaling [19]. In this context, EGCG plays a key role in the inhibition of proliferation and prompts apoptosis in an amount of cancer cell lines. EGCG was established to induce apoptosis in cells of the examined neoplastic cell lines in a concentration-related manner. Furthermore, the effect of EGCG on normal cells of the HS line was seen to be much less prominent than effects exerted on sensitive neoplastic cells [20] (Table 1). EGCG prompted apoptosis, diminished mitochondrial membrane potential and endorsed the G_0_/G_1_ phase cell cycle arrest of liver carcinoma cells whereas such activity was not observed on non-cancerous liver cells. Also, EGCG-induced apoptosis of cancer cells was linked with a substantial decrease in Bcl-2 and NF-κB expression [21].

### 2.5. Tumor Suppressor Genes 

Tumor suppressor genes are important gene as they are involved in inhibition of cell division, DNA damage repair and stimulation of apoptosis (Figure 3). Consequently, altered tumor suppression genes function lead in the development and progression of cancer. However, normal functioning of tumor suppressor genes is crucial in the inhibition of tumor development and progression. Vijay S Thakur et al. 2012 reported that EGCG activates p53 in human prostate cancer cells [22]. EGCG plays crucial role in the inhibition of anchorage-independent growth of human lung cancer cells through upregulating p53 expression. Besides, EGCG action can considerably increase p53 stability and encourage nuclear localization of p53 [23]. EGCG is capable of decreasing proliferation and inducing the apoptosis of pancreatic cancer cells linked with the expression of PTEN. Moreover, EGCG subdues the expression of *p*-Akt and *p*-mTOR through PTEN to regulate the PI3K/Akt/mTOR pathway [24] (Table 1).

### 2.6. Cell Cycle

The uncontrolled cell cycle plays a crucial role in the development and progression of cancer. The simultaneous action of several cellular signaling pathways which control cell cycle and apoptosis, is an important strategy to control cancer cell proliferation and the growth and progress of a tumor [25]. Natural products or active compounds from medicinal plants show anti-tumor activity through the induction of cell cycle arrest. A pioneering study demonstrated that combined EGCG and cisplatin treatment showed a synergistic cytotoxic effect in biliary tract cancer cell lines. Furthermore, EGCG decreases the mRNA levels of innumerable cell cycle-related genes, but enhances the expression of the cell cycle inhibitor p21 and the apoptosis-related death receptor 5 [26] (Table 1). Moreover, EGCG induces apoptosis in various ways including modulating pro- and anti-apoptotic proteins and cell cycle regulator proteins [27].

**Table 1:** Mechanism of action of EGCG in management of cancer through modulating cell signaling pathways.

Pathogenesis	Types of Genes	Mechanism	Refs.
Inflammation	Necrosis factor (TNF)-α/intercellular adhesion molecule-1 expression	EGCG protected against tumor necrosis factor-α-mediated lung inflammation through down-regulation of oxidative stress and intercellular adhesion molecule-1 expression	[5]
Breast cancer	Vascular endothelial growth factor	EGCG have been proven to reduce vascular endothelial growth factor production	[14]
Breast cancer	HIF-1α and NFκB	EGCG inhibited the activation of HIF-1α and NFκB and VEGF expression	[16]
Laryngeal carcinoma cells/ Colon carcinoma cells/Cervical carcinoma cells	Apoptosis	EGCG was found to induce apoptosis in cells of the examined neoplastic cell lines in a dose-related manner	[20]
Hepatocellular carcinoma	Bcl-2 and NF-κB	EGCG-induced apoptosis of cancer cells was linked with a substantial decrease in Bcl-2 and NF-κB expression	[21]
Human prostate cancer	p53	Epigallocatechin-3-gallate, activate p53 via acetylation at the Lys373 and Lys382 residues through inhibiting class I HDACs	[22]
Lung cancer	p53	EGCG play crucial role in the inhibition of anchorage-independent growth of human lung cancer cells through upregulating p53 expression	[23]
Pancreatic cancer	Pten	EGCG is capable of decreasing proliferation and induce the apoptosis linked with the expression of PTEN.	[24]
Pancreatic cancer	Pten	EGCG upregulate PTEN expression and downregulate the expression of pAKT and *p*-mTOR	[28]
Pancreatic cancer	PI3K/Akt/mTOR pathway	EGCG subdue the expression of *p*-Akt and *p*-mTOR through PTEN to regulate the PI3K/Akt/mTOR pathway	[24]
Biliary tract cancer	p21	EGCG reduced the mRNA levels of various cell cycle-related genes, but enhanced the expression of the cell cycle inhibitor p21	[26]

### 2.7. Phosphatidylinositide-3-Kinases (PI3Ks)/AKT Pathways 

PI3Ks/AKT pathways are over-activated in several types of cancers. Herbs and the active compounds of herbs show anti-tumor activity through the inhibition of the PI3/Akt/mTOR pathway. EGCG decrease proliferation and prompt apoptosis in a time- and dose-dependent manner and increase the PTEN expression and decrease the expression of pAKT and *p*-mTOR [28]. EGCG and whole green tea extract efficiently inhibited the accumulation of hypoxia-induced HIF-1α protein by delaying PI3K/Akt and ERKs signal pathways, and promoting the degradation of HIF-1α protein through the proteasome [29]. Remarkably, EGCG powerfully inhibited the basal activation of phospho-AKT and AKT kinase activity as early as 30 min after treatment. Also, inhibition of AKT kinase activity by EGCG headed the suppression of survivin, surveyed by increased caspase-9 activity. A dominant negative AKT or the phosphatidylinositol 3- kinase inhibitor, LY294002, powerfully inhibited surviving promoter activity, giving additional indications to support the hypothesis that the inhibitory effect of EGCG on *survivin* is arbitrated through the AKT pathway [30]. EGCG played a role in the induction of apoptosis and proliferation inhibition. The downregulated expressions of phosphorylated (*p*)-AKT and *p*-mTOR were partly weakened in PTEN-knockdown cells. Moreover, a study showed that EGCG can suppress the expression of *p*-AKT and *p*-mTOR via PTEN to regulate the PI3K/AKT/mTOR pathway [24]. 

### 2.8. Signal Transducer and Activator of Transcription 3 (STAT3)

STAT3 is one type of oncogene, which endorses cell survival, proliferation, motility, and progression in cancer cells [31]. EGCG plays an important role in cancer prevention by inhibiting the activity of Signal Transducer and Activator of Transcription 3 (STAT3). In this context, a previous finding revealed that EGCG, an active compound of green tea, plays a vital role in the suppression of the growth, invasion, and migration of pancreatic cancer cells, and the induction of apoptosis via interfering with the STAT3 signaling pathway [31]. Another study result demonstrated that the Stat3-binding assay showed that EGCG meaningfully disturbed Stat3 peptide binding at micromolar concentrations, and the docking experiments showed that EGCG had a powerful relation with Arg-609, one of the chief residues in the STAT3 SH2 domain that it important for Stat3 and phosphorylated peptide binding. Moreover, another study proposed that the anticancer function of green tea is a result of the inhibition of the STAT3 signaling pathway via EGCG [32]. In addition, curcumin in combination with EGCG reduced the tumor CM-induced transition of normal endothelial cells toward tumor endothelial cells through inhibiting JAK/STAT3 signaling pathway [32].

### 2.9. Epidermal Growth Factor Receptor (EGFR)

EGFR altered activity has been noted in various pathological conditions. However, its regulation is an important step in the inhibition of cancer. In this regard, EGCG shows a pivotal role in the inhibition of EGFR activity. The effects of epigalocathechin-3-gallate on the activation of the HER-2 receptor in human head and neck squamous cell carcinoma and breast carcinoma cell lines that show constitutive activation of HER-2 was investigated. Treatment of cells with 10 or 30 micrograms of epigalocathechin-3-gallate showed a 50% inhibition of growth, and decidedly inhibited the phosphorylation of HER-2 in both tested cell lines. Moreover, epigalocathechin-3-gallate inhibits activation of the epidermal growth factor receptor in carcinoma cells, and it was proposed that epigalocathechin-3-gallate may be valuable in treating cases of breast carcinoma and human head and neck squamous cell carcinoma in which activation of the EGFR and/or HER-2 plays significant roles in tumor survival and growth [33]. The causal mechanism of EGCG antitumor potency was mostly dependent on suppression of the epidermal growth factor receptor signaling pathway. Short-duration EGCG exposure considerably decreased EGF-induced EGFR, AKT, and ERK1/2 activation. Furthermore, long standing EGCG treatment inhibited total and membranous EGFR expression, decidedly reducing epidermal growth factor receptor nuclear localization and expression of the downstream target gene cyclin D1, showing that EGCG treatment suppressed epidermal growth factor receptor transactivation. Therefore, inhibition of the epidermal growth factor receptor signaling pathway may partially add to the anticancer activity of EGCG [34].

### 2.10. Activating Protein-1 (AP-1) 

Activating protein-1 transcription factor has been associated with pathogenesis including cancer. EGCG, an active compound of green tea, plays vital role in the inhibition of activating protein-1 (AP-1) transcription factor. Epigallocatechin gallate and theaflavins are supposed to be important active constituents in tea for the chemoprevention against cancer. EGCG and theaflavins inhibited epidermal growth factor- or 12-*O*-tetradecanoylphorbol-13-acetate-induced cell transformation in a dose-dependent manner. With a dose of 5–20 micro mol/L to inhibit cell transformation, Epigallocatechin gallate and theaflavins inhibited AP-1-dependent transcriptional activity and DNA binding activity. The inhibition of AP-1 activation occurs via the inhibition of a kinase-dependent c-Jun NH2-terminal [35].

### 2.11. Phase II Detoxifying Enzymes

The mechanisms of chemical protection against carcinogenesis and other forms of toxicity arbitrated through electrophiles is the initiation of enzymes involved in their deactivation, mainly phase II xenobiotic-metabolizing enzymes [36,37]. Numerous natural products or active compounds play a significant role in cancer prevention through inhibition of phase I enzymes and activation of phase II enzymes. Green tea polyphenol extract induces chloramphenicol acetyltransferase activity in human heptoma HepG2 cells transfected with a plasmid construct, which contains an antioxidant-responsive element and a minimal glutathione S-transferase Ya promoter linked to the CAT reporter gene. This indicates that GTP stimulates the transcription of Phase II detoxifying enzymes through the antioxidant-responsive element. Finally, action of GTP and the proposed stimulation of MAPKs may be possible signaling pathways utilized through GTP to activate antioxidant-responsive element-dependent genes [38]. At low concentrations of EGCG, activation of MAPK leads to antioxidant response element-mediated gene expression, such as phase II detoxifying enzymes. However, at higher concentrations of EGCG, continued activation of MAPKs including JNK leads to apoptosis [39].

## 3. Role of EGCG in Inhibition and Prevention of Various Types of Cancer

**Figure 4:**
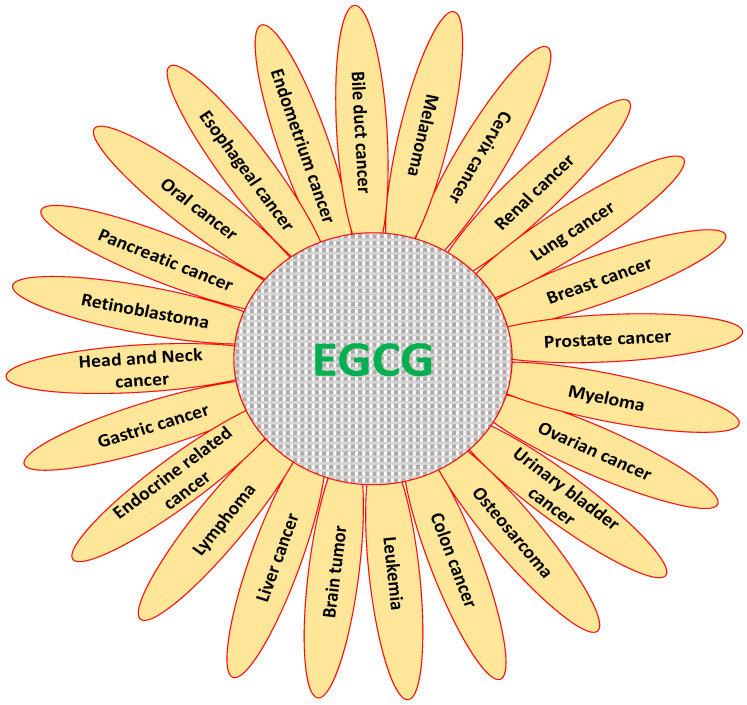
Role of EGCG in inhibition of numerous types of cancers.

Various types of herbal products compounds have been used as inhibitors of carcinogenesis process and act on various genes to regulate cancer growth [40,41]. EGCG plays pivotal role in the regulation of various types of cancer (Figure 4). The induction of cancer cell apoptosis and inhibition of carcinogenesis is the chief concern in anticancer compound research. The specific role of EGCG in prevention of various cancers based on in vitro studies are discussed here.

### 3.1. Cervix Cancer

EGCG is known for its antioxidant effects, and it functions as an antiproliferative agent. Furthermore, there is evidence that EGCG functions synergistically against cancer cell proliferation in joint treatment with anticancer drugs such as enoxacin [42]. Based on some results, this study has suggested that enoxacin and EGCG may be a valued treatment for cervical cancer [42] and EGCG might have suppressive effects on cervical cancer [43]. Debolina Pal et al. 2018 examined the antitumor activity of this compound and amarogentin either alone or in mixture. It was shown that EGCG with eugenol-amrogentin could more effectively constrain the cellular proliferation and colony formation than individual actions. Induction of apoptosis was also higher after treatment with EGCG in combination with eugenol-amrogentin than individual compound treatments [44]. Time-dependent exposure to Epigallocatechin gallate resulted in the reactivation of known tumor-suppressor genes in cancer cells due to marked changes in the methylation of the promoter regions of these genes. Overall, the existing study recommends that Epigallocatechin gallate may have a vital effect on the development of innovative epigenetic-based therapy [45]. Anti-angiogenic effects of epigallocatechin gallate treatment on tumor cells by assessing the alterations in the expression design of genes that are recognized to be involved in the angiogenesis process were investigated. The results have shown that Epigallocatechin gallate treatment causes down regulation of genes involved in the stimulation of adhesion, proliferation invasion and motility processes, and also play a lead role in the enhancement of numerous genes identified as having antagonist effects [46]. 

### 3.2. Breast Cancer

The potentiality of Epigallocatechin gallate to destroy breast cancer cell growth in vitro would be an indispensable mechanism. The effect of Epigallocatechin gallate on the metabolism of glucose was investigated. Epigallocatechin gallate reduced breast cancer cell growth. Besides, Epigallocatechin gallate decreased the expression of hypoxia-inducible factor 1α and glucose transporter-1, act as serious players in regulating glycolysis [47]. Chao-You Huang et al. reported that EGCG inhibited the proliferation of breast cancer cells in a concentration-dependent manner. The expression of P53 in the Epigallocatechin gallate -combined si-P53 group was greater than that of the si-P53 group, but less than the Epigallocatechin gallate group [48]. Another study was made to define the role of ER-α36 in the growth inhibitory activity of Epigallocatechin gallate towards ER-negative breast cancer cells. The result reported that Epigallocatechin gallate powerfully inhibited the growth of cancer stem/progenitor cells [49]. Moreover, Epigallocatechin gallate with dose of 80 μM considerably increased the genes of PTEN and decreased the Akt around equal to tamoxifen. In gene expression, Epigallocatechin gallate with the same dose meaningfully increased Bcl-2/Bax ratio. In protein expression of Bax/Bcl-2, Epigallocatechin gallate significantly increased six times while this ratio augmented ten-fold in the tamoxifen group [50]. The cell growth resulted in a dramatic decrease in breast cancer cell treated with various quantities of Epigallocatechin gallate, compared with control cells. Furthermore, protein expression of HIF-1α and vascular endothelial growth factor dropped in a dose-dependent manner in cancer cells pre-treated with increasing concentrations of Epigallocatechin gallate [51].

### 3.3. Ovarian Cancer

An experiment was made to assess the outcome mixtures of cisplatin with curcumin and Epigallocatechin gallate in ovarian cancer cell lines. Adding cisplatin before curcumin and Epigallocatechin gallate caused the greatest synergistic outcomes. When sequenced mixtures of cisplatin with curcumin and with Epigallocatechin gallate are given to ovarian cancer cell lines, lower concentrations and a smaller time gap between the two additions cause superior cytotoxic effects [52]. Inhibition of cell proliferation and the induction of apoptosis through Epigallocatechin gallate in the ovarian cancer cell line, and the effect of this compound on AQP5 expression, was checked. With growing concentrations of Epigallocatechin gallate and long treatment times, the growth inhibition rate of cancer cells slowly increased [53]. Addition of Epigallocatechin gallate improved the toxicity of cisplatin and EGCG increased cisplatin strength in cancer cell lines [54]. A study based on finding provide a novel insight into the mechanism by which EGCG, affecting multiple ET(A)R-dependent pathways, may inhibit ovarian carcinoma growth, proposing that EGCG may be beneficial in inhibiting as well as treating ovarian carcinoma in which ET(A)R activation by ET-1 plays a valuable role in tumor growth as well as progression [55]. EGCG plays an important role in decreasing ovarian cancer cell growth. Correspondingly, Epigallocatechin gallate showed growth inhibitory effects in each cell line in a dose-dependent approach and induced apoptosis and cell cycle arrest [56]. Besides, Epigallocatechin gallate causes a substantial decrease in ovarian cancer cell growth, showed dose dependent growth inhibitory effects, and induced apoptosis and cell cycle arrest. Moreover, the cell cycle was arrested at the G_1_ phase by EGCG in cancer cells [57].

### 3.4. Endometrium Cancer

Natural products have proven their anti-cancerous effect through modulating biological activities [58,59,60]. A recent study was performed to investigate how the treatment with Pro-Epigallocatechin gallate inhibited tumor angiogenesis in endometrial cancer. The results have revealed that Pro- Epigallocatechin gallate played a role in the inhibition of tumor angiogenesis in xenograft animal models via down-regulation of vascular endothelial growth factor A and HIF-1 α in tumor cells. vascular endothelial growth factors A secretion from endometrial cancer cells was decreased through Pro-Epigallocatechin gallate treatment [61]. Decreased estrogen and progesterone receptor expression was seen in Epigallocatechin gallate -treated endometrial adenocarcinoma, and decreased MAPK signals and phospho-Akt were noticed as well. Moreover, Epigallocatechin gallate caused the arrest of cells in the G_0_/G_1_ phase of the cell cycle [62]. Evidence showed that EGCG was established to inhibit proliferation in Ishikawa and in endometrial adenocarcinoma cells and efficiently decrease the expression of proliferation markers. This compound also suppressed the activation of ERK and downstream transcription factors fos and jun [63]. 

### 3.5. Pancreatic Cancer

Various studies provide evidence that this natural compound acts as an anti-cancer therapy through modulating various biological activity and killing cancerous cells and pathological conditions [64,65,66]. Epigallocatechin gallate inhibits pancreatic cancer cell migration and invasion. Epigallocatechin gallate plays a starring role in the inhibition of pancreatic cancer cell migration and invasion and the causal mechanisms for this were examined. This compound decreased pancreatic cancer cell migration, invasion and growth. Also, growth synergized with gemcitabine to decrease pancreatic cancer cell growth, migration, and invasion [67]. A recent study explored the effect and mechanism of action of growth alone and in mixture with chemotherapeutics on pancreatic cancer cell growth, focused on the metabolism of glycolysis. The results demonstrated that this catechin reduced pancreatic cancer cell growth, and the growth inhibition effect was further increased under glucose deficiency conditions [68]. Epigallocatechin gallate and Bleomycin, an anti-cancer chemotherapeutic drug, has improved anti-proliferative effects in vitro via induction of apoptosis of pancreatic cancer cells. Such a combination might show a new strategy with potential benefits for the treatment of pancreatic cancer [69]. The combination of the thermal cycling-hyperthermia and the chlorogenic acid or Epigallocatechin gallate significantly cause the anticancer effect against pancreatic cancer, whereas none of the single treatment induced such types of changes. The synergistic activity was credited to the cell cycle arrest at the G_2_/M phase and the induction of the reactive oxygen species-dependent mitochondria-mediated apoptosis [70]. A pioneering study was undertaken to evaluate how Epigallocatechin gallate targets the metabolism of pancreatic adenocarcinoma cells. It was reported that this catechin treatment of adenocarcinoma cells considerably reduced lactate production, anaerobic glycolysis, glucose consumption [71]. The molecular mechanisms of this molecule in human pancreatic cancer cells was investigated. The findings demonstrated that this green tea compound caused growth arrest at the G1 stage of the cell cycle, and induced apoptosis via the generation of reactive oxygen species [72].

### 3.6. Gastric Cancer

Gastric cancer-based studies have demonstrated that increasing the concentration of Epigallocatechin gallate inhibited cell proliferation under a hypoxia state and induced apoptosis in a dose-dependent manner [73]. Tang et al. reported that proliferation and tumor growth suppressed effects of this compound, on both the 5-fluorouracil resistant cells. Moreover, a remarkable finding demonstrated that reversal of 5-fluorouracil resistance of GC cells via Epigallocatechin gallate treatment. In the molecular study, it was conveyed that this green tea compound suppressed the expression of both MDR-1 and *p*-gp at mRNA and protein levels. Also, Epigallocatechin gallate was capable of inhibiting VEGF secretion and expression [74]. Epigallocatechin gallate significantly increased apoptosis and inhibited proliferation [75]. Chihiro et al. showed that Epigallocatechin gallate treatment of gastric cancer cells induced apoptosis, which was established by sub-G_1_ analysis, and EGCG treatment lowered surviving and increased Bax expression. [76]. In 2013, Jun Ma et al. investigated the inhibition of apoptosis and proliferation of undifferentiated gastric cancer cells, to form target genes for regulation by this green tea compound. Epigallocatechin gallate meaningfully promoted apoptosis and inhibited the proliferation of cancer cells [77]. The effect of this catechin on the growth of gastric cancer and its possible mechanism was examined. Findings revealed that intraperitoneal injection of this green tea catechin inhibited the growth of gastric cancer [78]. The inhibitory effect of this biomolecule on the growth and angiogenesis of gastric cancer was examined, and its molecular mechanism was explored. The results have shown that intraperitoneal injection of this biomolecule meaningfully inhibited the growth of gastric cancer. Moreover, secretion and mRNA expression of vascular endothelial growth factor in tumor cells were also suppressed by this compound in a dose-dependent manner [79].

### 3.7. Liver Cancer

A study based on liver cancer provided evidence that Epigallocatechin gallate, due to its anti-cancer effect, might be an efficient target of catechin in liver carcinoma [80]. In 2016, Subhayan Sur et al. evaluated chemopreventive and therapeutic efficacy of this green tea compound and theaflavin on self-renewal Wnt and hedgehog pathways. Both Epigallocatechin gallate and theaflavin limited the development of hepatocellular carcinoma of carcinogen application showing potential chemoprevention in the continuous treated group followed by pre-treated and therapeutic effectiveness in the post-treated group. This restriction was linked with a considerable decrease of proliferation, increased apoptosis, decreased prevalence of hepatocyte progenitor cell and stem cell population, irrespective of EGCG/theaflavin treatments [81]. Another pioneer study reported that EGCG, an active compound of green tea reduced hypoxia-incited apoptosis in HepG2 cells as well as enhanced cell survival. These finding support the hypothesis that EGCG might be measured a useful agent for hepatocellular carcinoma treatment [82]. An interesting finding in the favor of this catechin role in liver cancer reported that this compound increased the animal survival and decreased both α-fetoprotein and HepG2 viability [83]. 

### 3.8. Colon Cancer

Another study result showed that the sensitivity of colon cancer cells to methylation shows a vital effect in its response to alternative therapy concerning epigallocatechin gallate [84]. The role of the combinatorial effects of this green tea compound and sodium butyrate in the regulation of survivin, which is an overexpressed anti-apoptotic protein in colon cancer cells, was evaluated. The results reported that the mixture treatment induced apoptosis and cell cycle arrest in colorectal cancer cells. Also, G2/M arrest was seen for cancer cells, and G1 arrest for colorectal cancer cells for combinatorial treatment [85]. Another study was made to explore the mechanism of causal Epigallocatechin gallate-induced downregulation of epidermal growth factor receptor in colon cancer cells [86]. Both Epigallocatechin gallate and Poly E specially inhibited the growth of the various colon cancer cells when compared with the normal human fetal colon cell line. Moreover, treatment of cancer cells with Epigallocatechin gallate or Poly E caused an increase of cells in G1 and induced apoptosis. Both Epigallocatechin gallate and Poly E initiated a decrease in the phosphorylated forms of epidermal growth factor receptor and HER2 proteins, and successively caused a decrease in the phosphorylated forms of the extracellular signal-regulated kinase and Akt proteins [87]. Compared with the positive control group, Epigallocatechin gallate treatment dependently decreased tumor load per mouse [88]. A pioneering study was made to examine whether this compound could prevent the occurrence or metastases of orthotopic colon cancer and probe the emphasized mechanisms. The results showed that inhibition of Epigallocatechin gallate on growth and metastases of colon tumor implanted orthotopically in the cecum of nude mice. The inhibition rates on tumor growth in the EGCG groups were significantly different compared with the control group. In addition, different doses of this green tea compound were able to inhibit liver and pulmonary metastases to varying degrees [89]. 

### 3.9. Bile Duct Cancer

Anticancer activities of epigallocatechin gallate against cholangiocarcinoma cells was examined. This compound efficiently inhibited the growth of cancer cells and induced apoptotic cell death [90]. The effects of Quercetin on Janus-like kinase/signal transduction and transcription pathway of cholangiocarcinoma was investigated. It was seen that the Janus-like kinase/signal transduction and transcription pathway activation through proinflammatory cytokine in cancer cells was decreased through pretreatment with quercetin and epigallocatechin gallate [91]. A recent study based on cholangiocarcinoma was performed to investigate the effect of combination of vorinostat and epigallocatechin gallate against cholangiocarcinoma cells. The combination of vorinostat and this green tea compound revealed synergistic growth inhibitory effects and caused stimulation of apoptosis in tumor cells [92]. Apoptosis was increased in cells incubated with this catechin and GEM [93].

### 3.10. Renal Cancer

Findings based on renal cancer revealed that Epigallocatechin gallate inhibit growth and induces apoptosis in the renal cell carcinoma cell line. Moreover, western blotting and real-time PCR-based results confirm that this catechin plays a role in the increase of the expression of TFPI-2. Besides, findings advocate that this green tea compound inhibits growth and induces apoptosis [94]. Epigallocatechin gallate showed the potential to inhibit the proliferation of carcinoma cells, induce apoptosis and efficiently suppress the migration and invasion of renal cell carcinoma cells. Moreover, Epigallocatechin gallate treatment caused in the downregulation of metalloproteinase-2 and metalloproteinase-9 in carcinoma cells and anticancer effect associated with Epigallocatechin gallate may involve the downregulation of metalloproteinase-2 and metalloproteinase-9 [95]. Epigallocatechin gallate showed the potential to inhibit the proliferation of carcinoma cells, encourage apoptosis and efficiently suppress the migration and invasion of renal cell carcinoma cells. Moreover, Epigallocatechin gallate treatment caused the downregulation of metalloproteinase-2 and metalloproteinase-9 in renal cell carcinoma cells, and anticancer effects associated with EGCG may involve the downregulation of matrix metalloproteinase-2 and 9 [95]. Green tea extract strongly inhibited the growth of both RCC cell lines in a concentration-dependent manner [96]. A novel study was performed to examine if this catechin could enhance susceptibility of carcinoma cells to vinblastine. Epigallocatechin gallate treatment provoked important upregulation of Cx32. Chemical sensitivity to vinblastine in cells was increased by EGCG pre-treatment, and this effect was withdrawn by siRNA-mediated knockdown of connexin 32 [97].

### 3.11. Prostate Cancer

Green tea catechins, epicatechin, epigallocatechin and epigallocatechin-3-gallate were examined in the regulation of androgen receptor acetylation in androgen-dependent prostate cancer cells. Green tea compounds induced prostate cancer cell death, suppressed agonist-dependent androgen receptor activation and AR-regulated gene transcription [98]. A novel study based on a prostate cancer study reported that human prostate cancer cell lines are repressed by the green active compound epigallocatechin-3-gallate [99]. A pioneering study was made to validate the combined beneficial anticancer effects of curcumin and EGCG on prostate cancer cells, which are resistant to chemotherapy drugs and apoptosis inducers. Besides, this green tea compound demonstrated a lower inhibitory effect on cancer cell proliferation than prostate cancer cell lines. Co-treatment of curcumin enhanced antiproliferative effect of epigallocatechin-3-gallate on prostate cancer cells. The protein expressions of p21 were meaningfully increased by the co-treatment of EGCG and curcumin, while they were unchanged by the treatment with each compound alone [100]. The synergistic effect of epigallocatechin-3-gallate and ibuprofen was evidences in prostate cancer and it was demonstrated that epigallocatechin-3-gallate + ibuprofen treatment caused around 90% of growth inhibition [101]. EGCG showed anticancer effects, and it was proved that this catechin inhibited cancer cell proliferation in a concentration-dependent fashion. Besides, the treatment of prostate cancer cells with EGCG caused in time and concentration-dependent activation of the extracellular signal-regulated kinase pathway [102]. The inhibitory effects of green tea components, epigallocatechin-3-gallate, were tested on the prostate cancer cell lines. EGCG was demonstrated to be the greatest powerful catechin at preventing cell growth. The inhibition induced by EGCG was shown to increase through apoptotic cell death, as observed by changes in nuclear morphology and DNA fragmentation [103]. 

### 3.12. Urinary Bladder Cancer

The therapeutic implication of epigallocatechin-3-gallate in urinary bladder was confirmed through antiproliferation and antimigration effects against bladder cancer. It was reported that treatment with this green tea compound caused an important inhibition of cell proliferation via induction of apoptosis, without noticeable toxicity to normal bladder epithelium cells. Epigallocatechin-3-gallate also inhibited cancer cell invasion and migration, and Epigallocatechin-3-gallate induced apoptosis in cancer cells [104]. A pioneering study was performed in consideration of role of this green tea catechin on the estimation of Epigallocatechin-3-gallate-induced apoptosis in bladder carcinoma cells and its related molecular mechanisms. Based on an in vitro study, Epigallocatechin-3-gallate caused morphological changes and increased growth inhibition in a dose- and time-dependent manner in bladder cancer cells. Additionally, sub-G1 populations were revealed and caspase3 and -9 activities were stimulated in Epigallocatechin-3-gallate -treated bladder cancer cells [105]. This compound showed a role in the inhibition of bladder carcinoma cell growth and decreased the in vitro migration capacity of cells through downregulation of *N*-cadherin and inactivation of Akt signaling [106]. The ability of epigallocatechin-3-gallate in comparison with mitomycin to prevent tumor cell implantation/growth in an animal model of superficial bladder cancer was investigated. Results demonstrate that Epigallocatechin-3-gallate and mitomycin C inhibited intravesical tumor growth in a concentration and time-dependent fashion [107].

### 3.13. Leukemia 

Epigallocatechin-3-gallate has proven its ability to inhibit acute leukemia cell proliferation and apoptosis as this compound caused anti-cancerous epigenetic changes [108]. The effects of this catechin on proliferation and cell cycle of acute promyelocytic leukemia cell line was investigated. The result showed that proliferation and cell cycle progression of leukemia cells treated with EGCG were inhibited and demonstrated time-dependent and dose-dependent characteristics [109]. Epigallocatechin-3-gallate caused significant inhibition of proliferation and induced apoptosis in cancer cells. This effect was linked with decreased expressions of multidrug resistant proteins whereas the expressions of pro-apoptotic genes was significantly increased [110]. Moreover, another study demonstrated that epigallocatechin-3-gallate treatment meaningfully inhibited the viability of leukemia cells in a dose-dependent way. Besides, Epigallocatechin-3-gallate treatment induced apoptosis and increased the levels of Bax protein expression [111]. Study results reported that green tea compound targets PML/RARα oncoprotein in the degradation and potentiates differentiation of leukemia cells in combination with ATRA via PTEN [112]. A growth decrease and apoptosis prompting effect of EGCG and epigallocatechin were examined against leukemia cells. Epigallocatechin-3-gallate showed higher growth decrease against cancer cells than epigallocatechin and both compound of green tea prompted apoptosis demonstrated via nuclei fragmentation and nuclear fragmentation [113]. 

### 3.14. Lymphoma 

The effect of epigallocatechin-3-gallate on proliferation and apoptosis in the B lymphoma cell lines was investigated. The result confirmed that epigallocatechin-3-gallate induced growth inhibition and apoptosis [114]. The effect of this green tea catechin alone or in combination with trichostatin A in malignant lymphoma cells was examined. Both epigallocatechin-3-gallate and trichostatin alone inhibited lymphoma cell proliferation; the collective treatment meaningfully reduced cancer cell proliferation. Cells treated with epigallocatechin-3-gallate or the mixture treatment had lower proliferative indices when compared to the other groups. The outcome verified that, in comparison with the control, changed concentration of epigallocatechin-3-gallate were capable of preventing the growth of cancer cell lines and increase the cell number in G0/G1 phase. After treatment with this catechin, the methylation level was apparently decreased [115]. The role of epigallocatechin-3-gallate on lymphoma cells in cell death was evaluated. Results demonstrated that epigallocatechin-3-gallate caused induction of cell death and reactive oxygen species generation in lymphoma cells [116]. 

### 3.15. Head and Neck Cancer

The mechanism that epigallocatechin-3-gallate inhibits the growth of head and neck cancer, focused on the regulation of the expression and activity of β-catenin showed epigallocatechin-3-gallate prompted apoptosis via the suppression of β-catenin signaling [117]. The anti-tumor effect of epigallocatechin-3-gallate on head and neck cancer stem cells demonstrated that this green tea catechin inhibits the self-renewal capacity of carcinoma cancer stem cells through subduing their sphere forming capacity. Additionally, epigallocatechin-3-gallate treatment increased cisplatin-mediated chemosensitivity, and mixture treatment of this compound and cisplatin decrease tumor formation and encouraged apoptosis in a xenograft model [118]. A pioneering study explaining the role epigallocatechin-3-gallate in cancer management as shRNA-mediated silencing of Bim meaningfully inhibited apoptosis prompted through the combination of erlotinib and epigallocatechin-3-gallate [119]. The treatment of carcinoma cell lines with simultaneous treatment with decreased cell proliferation and erlotinib strongly inhibited erlotinib-induced expression of p21 and p27. Furthermore, erlotinib increased the expression of p53 protein, the ablation of which by short hairpin RNA powerfully inhibited decreased cell proliferation and erlotinib-mediated growth inhibition [120]. 

### 3.16. Oral Cancer

The therapeutic potential of epigallocatechin-3-gallate for aiming oral squamous cell carcinoma reported that epigallocatechin-3-gallate compound suppressed cancer cells viability. Cell cycle analysis confirmed that this catechin induced G1 phase arrest of the tumor cells and treatment with this compound meaningfully increased caspase-7 and -3 activities, and the percentage of apoptotic cells when compared with control cells [121]. Anticancer activity of Epigallocatechin-3-gallate was confirmed as this compound inhibited cell viability and induced CAR cell apoptosis and autophagy. Epigallocatechin-3-gallate also considerably enhanced caspase-3 and caspase-9 activities and evidently increased the protein levels of Bax, cleaved caspase-9, cleaved caspase-3, in cancer cells. Significantly, the protein and gene expression of multidrug resistance were dose-dependently inhibited by this compound [122]. Epigallocatechin-3-gallate, the main component of green tea, has been shown to to considerably inhibit the expression of indoleamide 2,3-dioxygenase, which is induced by gamma-interferon in cancer cell lines. Besides, Epigallocatechin-3-gallate suppresses the induction of indoleamide 2,3-dioxygenase at transcriptional level [123]. The effects of epigallocatechin-3-gallate in inhibiting HGF-induced tumor growth and invasion of oral cancer in vitro was confirmed through this compound pointedly inhibited HGF-induced phosphorylation of Met and invasion, cell growth, and expression of MMP-9 and 2 [124]. A recent study explained the molecular confirmation associated with the antimetastatic effect of this catechine in an oral squamous cell culture system through presenting an approximately complete inhibition on the invasion cancer cells via a decreased expression of matrix metalloproteinase-2. Moreover, epigallocatechin-3-gallate caused an inhibitory effect on cell migration, motility, spread, and adhesion [125].

### 3.17. Oesophagus Cancer

Epigallocatechin-3-gallate inhibited the growth of oesophageal cancer and induced apoptosis, decreased the bcl-2 protein expression and increased the expression of caspase-3 and Bax protein [126]. A curcumin, epigallocatechin-3-gallate and lovastatin-based study reported that these drugs alone or in combination considerably reduced the invasion and viability capacity of cancer cells in vitro. Moreover, these compounds reduced the expression of and cyclooxygenase-2phosphorylated extracellular-signal-regulated kinases. Besides, the nude mouse xenograft assay showed that epigallocatechin-3-gallate and the combinations of curcumin, epigallocatechin-3-gallate and lovastatin suppressed cancer cell growth [127]. An oesophageal squamous cell carcinoma-based result demonstrated that epigallocatechin-3-gallate inhibited proliferation cancer cells. Besides, tumor cells were arrested in the G1 phase and apoptosis was convoyed by ROS production and caspase-3 cleavage [128]. The effects of the combination of EGCG or TF3 with Vc on the apoptosis and caspases-9/3 activities in lung adenocarcinoma cells and oesophageal carcinoma were examined. The results demonstrated that Vc enhanced the epigallocatechin-3-gallate and TF3 induced apoptosis of cancer cells, and this effect involved the activation of caspase-9 and 3 [129]. 

### 3.18. Melanoma

Vorinostat alone or in combination with Epigallocatechin-3-gallate imparts anti-proliferative effects against melanoma cells as evidences anti-proliferative effects of vorinostat were better than or similar to those of Epigallocatechin-3-gallate among the cell lines tested. Besides, the mixture treatment resulted in a meaningful inhibition of cell proliferation, superior increased apoptosis, activation of p21, p27 and caspases and Bax and Bcl2 protein [130]. Epigallocatechin-3-gallate and/or Interferon-α 2b treatments to melanoma cells caused in a noticeable decrease in cell proliferation and caused induction of apoptosis. Remarkably, the mixture was noticed to be more effective than either of the agents alone [131]. Epigallocatechin-3-gallate was shown to have a vital role in the inhibition of melanoma cell growth at physiological doses and reduced NF-κB activity was linked with decreased IL-1β secretion from melanoma cells [132]. Anti-metastatic effects of this compound or the combination with EGCG and dacarbazine on melanoma cells in vitro demonstrated that epigallocatechin-3-gallate inhibited melanoma cell migration and invasion. Moreover, epigallocatechin-3-gallate significantly inhibited the tyrosine phosphorylation of focal adhesion kinase and the activity of matrix metalloproteinase-9. In animal experiments, the result showed that epigallocatechin-3-gallate alone reduced lung metastases in mice bearing melanomas [133]. The inhibitory effects of epigallocatechin-3-gallate on the invasion of malignant melanoma cell line was noticed as this compound was suggested to enhance the expression of E-cadherin. It was recommended that this green tea compound powerfully inhibited invasion of cancer cells, and the inhibition mechanism was probably related to the upregulation of E-cadherin expression [134].

### 3.19. Lung Cancer

Metformin inhibited HO-1 expression and increased the anti-tumor effect of epigallocatechin-3-gallate and metformin also enhanced ROS generation induced by epigallocatechin-3-gallate, consequently causing apoptosis [135]. It was reported that hsa-mir-485-5p was decreased in serum samples from patients with cancer cells. In the meantime, epigallocatechin-3-gallate played a role in the enhancement of hsa-mir-485-5p expression and Hsa-mir-485-5p mimics noticeably induced cell apoptosis and inhibited cancer cell growth [136]. The treatment of cancer cells with epigallocatechin-3-gallate -induced apoptosis through increased the expression of cleaved caspase-3 and Bax, whereas decreased the expression of Bcl-xL [137]. Tumorsphere formation assay was used to improve lung cancer stem cells. It was demonstrated that Wnt/β-catenin pathway was activated in lung cancer stem cells, and the downregulation of β-catenin. Moreover, epigallocatechin-3-gallate efficiently reduced lung cancer stem cells’ activity through decreasing lung cancer stem cell markers, inhibiting tumorsphere formation, decreasing proliferation and encouraging apoptosis [138]. The cytotoxic effect of this green tea compound on both the parental lung cancer cells and their cisplatin-resistant cells explained that epigallocatechin-3-gallate was capable of increasing interlukine-6 production, while its downstream effector signal transducers and activators of transcription 3 phosphorylation were unaffected by epigallocatechin-3-gallate [139].

### 3.20. Myeloma

The effect and fundamental mechanism of epigallocatechin-3-gallate on multiple myeloma cells confirmed that this green tea compound decreased proliferation and induced apoptosis in cancer cells [140]. The activity of EGCG against the myeloma cell line was investigated. The outcome of the study revealed that the treatment of the cancer cell line with EGCG inhibits cell proliferation and induces apoptosis, and a synergistic effect was observed when EGCG and bortezomib are combined [141]. The effect of EGCG on angiogenesis induced by multiple myeloma cell line cancer cell and its mechanism was evident by epigallocatechin-3-gallate inhibiting the effect of endothelial cell migration induced by cancer cell supernatant, and the numbers of migrated cells and numbers of migrated cells showed negative correlation with the dose of epigallocatechin-3-gallate [142]. EGCG has proven its role in the induction of both dose- and time-dependent growth arrest and following apoptotic cell death in multiple myeloma cell lines such as IL-6-dependent cells and primary patient cells [143].

### 3.21. Osteosarcoma

A study was performed to evaluate the effects of epigallocatechin-3-gallate on osteosarcoma cells and elucidate the primary mechanism in the management of osteosarcoma. The results based on cellular function assays showed that epigallocatechin-3-gallate compound induced cell cycle arrest, inhibited cell proliferation, caused induction of apoptosis, and inhibited the growth of tumors. Overall, the outcome of the study revealed that epigallocatechin-3-gallate has an anticancer effect on osteosarcoma cells [144]. The effects of miR-126 and epigallocatechin-3-gallate apoptosis, cell viability, cell cycle distribution of osteosarcoma cells was investigated. The results demonstrated that epigallocatechin-3-gallate showed role in the suppression of proliferation of osteosarcoma cells [145]. The effects of anti-inflammatory IL-1 receptor antagonist was evaluated individually and in combination with EGCG on the production of interlukin-1-induced tumorigenic factors in osteosarcoma cells. The result confirmed that IL-1Ra and epigallocatechin-3-gallate downregulated interlukin-1-induced interlukin-6 and interlukin-8 release from cancer cells. Interlukin-1Ra and epigallocatechin-3-gallate caused reduction of secretion of invasiveness-promoting matrix metalloproteinase-2 [146].

### 3.22. Brain Tumor

Epigallocatechin-3-gallate confirmed its role in the inhibition of glioma and it was reported that epigallocatechin-3-gallate induced apoptosis. Moreover, the mitogen-activated protein kinase pathway was shown to be involved in apoptosis and proliferation [147]. The role of this catechin in the inhibition of cancer was examined based on the glioblastoma cell. Treatment with subtoxic doses of epigallocatechin-3-gallate in combination with TRAIL induces rapid apoptosis, proposing that this combined treatment may offer an attractive strategy for treating gliomas [148]. Another study was performed to investigate the anticancer effect of epigallocatechin-3-gallate on the growth and invasive ability of glioma cells. To analyze the study, two glioma cell lines were treated with epigallocatechin-3-gallate, and its effect on cell proliferation and invasive ability. The study findings revealed that epigallocatechin-3-gallate treatment leads to a decrease in cell viability and the S-phase cell fraction. Besides, invasive ability was significantly suppressed in the EGCG-treated cells [149]. To know whether drinking green tea has chemopreventive effects on brain cancer cells, a study was performed to examine the effect of epigallocatechin-3-gallate on human glioblastoma cell cultures. Results confirm that after treatment EGCG, strong induction of autophagy and apoptosis was noticed [150].

### 3.23. Endocrine Related Cancer

In 2019, Dongdong Wu et al. reported that epigallocatechin-3-gallate, an active compound of green tea, inhibited the viability, proliferation and cell cycle progression in thyroid carcinoma cells [151]. Another study reported that epigallocatechin-3-gallate considerably suppresses invasion and migration in anaplastic thyroid carcinoma cells [152]. The effect of epigallocatechin-3-gallate on the proliferation and motility of thyroid papillary and follicular carcinoma cell lines was confirmed as epigallocatechin-3-gallate treatment inhibited the growth of carcinoma cells [153]. 

### 3.24. Retinoblastoma 

A study based on epigallocatechin-3-gallate (EGCG) was performed to measure the involvement of the retinoblastoma-E2F/DP pathway as an important contributor in the antiproliferative effects of epigallocatechin-3-gallate. The result showed that this green tea compound treatment of cancer cells resulted in a decrease in the total retinoblastoma with a relative increase in the hypophosphorylated form of pRb. Also, pRb was found to downregulate the protein expression of other members of the pRb family [154] (Table 2). 

## 4. In Vivo Efficacy of Epigallocatechin-Gallate (EGCG) in the Management of Cancer

The chemopreventive effects of EGCG have been also proven by in vivo experiments on several animal models. In this context, a pioneering study reported that mice treated with EGCG or sorafenib, effective anti-HCC drug in clinical practice alone showed a significantly smaller tumor diameter than untreated mice. On the other hand, EGCG combined with sorafenib meaningfully suppressed tumor size compared with sorafenib alone. Moreover, compared to the sorafenib alone, combination treatment with EGCG and sorafenib meaningfully increased the rate of apoptosis [155]. EGCG suppressed tumor growth based on an in vivo study through decreasing the expression of miR-25 and proteins linked with apoptosis, which was additional established by a reduction of Ki-67 and increase of pro-apoptotic PARP expression [156]. The effect of catechins on the antitumor efficacy of doxorubicin (DOX) in a murine model for chemoresistant hepatocellular carcinoma (HCC) was examined. It was reported that ECG or EGCG at higher doses had a minor inhibitory effect on cell proliferation in the resistant human HCC cell line and in vivo, whereas the administration of DOX with these compounds at lower doses significantly inhibited HCC cell proliferation in vitro and hepatoma growth in a xenograft mouse model, compared with treatment with either agent alone at the same dose [157]. The chemopreventive and therapeutic efficacy of tea polyphenols epigallocatechin gallete (EGCG) and theaflavin (TF) on self-renewal Wnt and Hedgehog (Hh) pathways during CCl4/*N*-nitosodiethylamine-induced mouse liver carcinogenesis was evaluated. The result of the study established that moderately increased body weights were noticed due to EGCG/TF treatment compared with carcinogen control mice. Both EGCG and TF could restrict the development of hepatocellular carcinoma at the 30th week of carcinogen application, showing potential chemoprevention in the continuous treated group followed by pretreated and therapeutic efficacy in the posttreated group [81]. Treatment of mice with EGCG showed a noteworthy decrease in the mean number of aberrant crypt foci per mouse, when compared with the model mice treated with azoxymethane (AOM)/dextran sodium sulfate (DSS). As compared with the positive control group, EGCG treatment dependently decreased tumor load per mouse by 85%. The results revealed that EGCG could inhibit colon carcinogenesis via decreasing the number of precancerous lesions and solid tumors, with reduced tumor load and delayed histological progression of colorectal cancer [88]. Epigallocatechin gallate prevents the occurrence or metastases of orthotopic colon cancer and the underlying mechanisms of this were investigated. Scientists recorded the inhibition of Epigallocatechin gallate on the growth and metastases of a colon tumor implanted orthotopically in the cecum of nude mice. Moreover, the finding revealed that Epigallocatechin gallate has a protective effect on the growth and liver and pulmonary metastases of orthotopic colon cancer in nude mice, and such anticancer results might be partially produced through activating the Nrf2-UGT1A signal pathway [89]. Green tea polyphenols were investigated as adjuncts to chemotherapy for cholangiocarcinoma. Results confirmed that Epigallocatechin-gallate decreased in vivo growth and increased the sensitivity to GEM of Mz-ChA-1 cell xenografts in nude mice [93]. Oral squamous cell carcinoma (OSCC) is one of the most common malignant tumors in the oral region. Despite current therapeutic strategies, the survival rate has not been improved for several decades. Thus, it is important to develop a novel approach for the treatment of OSCC. Epigallocatechin-3-gallate (EGCG) is a major constituent. The therapeutic potential of Epigallocatechin gallate (EGCG) for targeting human OSCC in vivo was evaluated. Results revealed that in an in vivo xenograft experiment on mice, Epigallocatechin gallate (EGCG) treatment resulted in a 45.2% reduction in tumor size as compared with the control group without weight loss. There were noteworthy differences in Ki-67 expression between the EGCG treatment group and control group, and the percentage of apoptotic cells in the Epigallocatechin gallate (EGCG) treatment group was meaningfully larger than that in the control group [121]. 

## 5. Measurement of Safety, Efficacy and Tolerability of EGCG in Cancer Based on Human Clinical Trials

A laboratory experiment provided evidence that Epigallocatechin-3-gallate modulates numerous molecular targets and inhibit the pathogenesis of cancer through inhibition of initiation, promotion and progression. Moreover, clinical human trial-based studies are still needed to establish the efficacy of Epigallocatechin-3-gallate in management of cancer. In this regard, various clinical trials on human subjects confirm that EGCG plays a role in cancer prevention.

### 5.1. Prostate Cancer

In a placebo-controlled, randomized clinical trial of Polyphenon E, a mixture of green tea catechine containing 400 mg epigallocatechin-3-gallate per day, was conducted on 97 men with high-grade prostatic intraepithelial neoplasia and/or atypical small acinar proliferation. The primary study endpoint was an assessment of the cumulative one-year prostate cancer rates on the two study arms. No differences in the number of prostate cancer cases were seen. This finding was determined by a decrease in atypical small acinar proliferation diagnoses on the Poly E (0/26) compared with the placebo arm (5/25). A decrease in serum prostate-specific antigen was observed on the PolyE arm. Daily consumption of a standardized, decaffeinated catechin mixture of 400 mg epigallocatechin-3-gallate per day for 1 year gathered in plasma and was well tolerated but did not reduce the possibility of prostate cancer [158]. Another study was conducted a placebo-controlled, randomized clinical trial of Polyphenon E, a branded mixture of decaffeinated catechine, comprising 0.4 g EGCG per day, in 97 men with high-grade prostatic intraepithelial neoplasia and/or atypical small acinar proliferation. PolyE with 200 mg epigallocatechin-3-gallate was given with food. A secondary study endpoint in this trial was a comparison of the overall one-year treatment related adverse events and grade 3 or higher adverse event on the two study arms. The monthly assessments of toxicity, concomitant medications and organ function were performed. Daily intake of a standardized, decaffeinated, catechin mixture having 200 mg Epigallocatechin-3-gallate, BID taken with food for 1 year accumulated in plasma and was well tolerated and did not show that the treatment was related to any adverse effects in men with baseline high-grade prostatic intraepithelial neoplasia or atypical small acinar proliferation [159]. 

### 5.2. Urinary Bladder Cancer

A pioneering study was performed as a phase II pharmacodynamic prevention trial of Polyphenon E (formulation mainly containing Epigallocatechin-3-gallate) in patients prior to bladder cancer surgery. The patients of bladder tumor were randomized to receive Polyphenon E containing either 0.8 g or 1.2 g of epigallocatechin-3-gallate or placebo for fourteen to twenty-eight days before the transurethral resection of a bladder tumor. There was not a noteworthy difference in Epigallocatechin-3-gallate tissue levels between two Polyphenon E dosage groups combined versus placebo. However, a dose-response relationship for Epigallocatechin-3-gallate levels was observed in both normal and malignant bladder tissue across the three study arms. In addition, EGCG levels in plasma and urine increased and clusterin were downregulated in a dose-dependent fashion. The epigallocatechin-3-gallate levels in plasma, urine, and bladder tissue followed a dose response association, as did modulation of tissue biomarkers of proliferation and apoptosis [160]. 

### 5.3. Head and Neck Cancer

A phase I study was performed to evaluate the safety and effectiveness of epigallocatechin-3-gallate mouthwash when given along with radiation in head and neck cancer. Head and neck cancer were enrolled in this study and Epigallocatechin-3-gallate mouthwash was administered at the assigned dosage level (starting at 440 micromol per liter and 3 times a day) in a standard 3 plus 3 dose escalation design. Mucosal toxicity, mucositis-related pain and patient satisfaction were evaluated weekly. The primary endpoint was safety of this green tea compound, and the secondary endpoint was to determine the relief of the mucositis symptom. The maximum tolerated dose of the Epigallocatechin-3-gallate mouthwash was 2200 micromol per liter. Burning and nausea were the most usual types of toxicities. Mucositis-related pain scores considerably decreased after epigallocatechin-3-gallate administration over time. Addition of this catechin mouthwash to radiotherapy is feasible without increasing toxicities. The recommended dose for phase II study is determined to be 1760 micrpmol per liter, and epigallocatechin-3-gallate administration decreased radiation-induced oral mucosal injury in patients [161].

### 5.4. Breast Cancer

The tolerability, safety and effectiveness of topical Epigallocatechin-3-gallate for radiation dermatitis of breast cancer patients getting adjuvant radiotherapy was investigated. The breast cancer patients which received radiotherapy to the chest wall after mastectomy were included. This green tea compound was sprayed to the radiation field from the beginning of Grade 1 radiation dermatitis for two weeks after finishing radiotherapy. Epigallocatechin-3-gallate concentration escalated from 40 to 660 micromol per liter in 7 levels with 3 to 6 patients in each level. Acute skin redness was noticed in 1 patient and measured to be related to the Epigallocatechin-3-gallate treatment at 140 micromol per liter level. Some patients included at this level did not experience toxicity to epigallocatechin-3-gallate. No other reported acute toxicity was related with Epigallocatechin-3-gallate. Grade 2 radiation dermatitis was noticed in eight patients during or after radiotherapy. The topical administration of Epigallocatechin-3-gallate was well tolerated and the maximum tolerated dose was not found [162]. 

### 5.5. Ovarian Cancer

A two stage, single arm, phase II study was performed to evaluate the safety and efficacy of Epigallocatechin-3-gallate enriched tea drink, as a treatment in women with advanced stage serous or endometrioid ovarian cancer. Women having FIGO stage III-IV serous or endometrioid ovarian cancer were enrolled in this study. They all had to drink 500 mL of the double-brewed green tea daily until recurrence or during a follow-up of 18 months. The primary endpoint was the absence of recurrence at eighteen months. During the first stage of the study, only five of the sixteen women remained free of recurrence eighteen months after complete response. Accordingly, the clinical trial was terminated. The women′s adherence to double brewed green tea was high, but six women stopped the interference before the end of their follow-up. No severe toxicity was noticed, but double-brewed green tea supplementation does not appear to be a promising maintenance intervention in women with advanced stage ovarian cancer after standard treatment [163].

### 5.6. Lung Cancer

Phase I study of Epigallocatechin-3-gallate in mishmash with normal chemoradiation in unresectable stage III lung cancer was investigated. Chemotherapy drugs such as cisplatin and etoposide were given concurrently with radiation. Moreover, Epigallocatechin-3-gallate solution was given three times a day after the occurrence of grade II esophagitis at six concentration levels and dose escalation, oesophageal toxicity and patient-reported pain was checked weekly. Patients were treated in six cohorts at six dose levels of Epigallocatechin-3-gallate. There were no dose limiting toxicities was noticed in all Epigallocatechin-3-gallate dosing tiers. Intense regression of esophagitis to grade 0/I was observed in 22 of 24 patients, while grade two esophagitis continued in 2 of 24 patients at the end of radiotherapy. Based on this result, it was concluded that the oral administration of Epigallocatechin-3-gallate is feasible, safe, and effective. The phase II recommended concentration is 440 micromol per liter [164].

## 6. Effect of EGCG Alone and in Combination with Other Anticancer Compounds or Related Molecules 

The chemotherapeutic drugs such as doxorubicin, cisplatin, 5-fluorouracil, and paclitaxel 6-mercaptopurine, cytarabine are commonly used to treat cancer but such drugs cause adverse side effects including fatigue, hair loss, infection, nausea and vomiting, appetite changes, and changes in the physiological and biochemical processes. The additive or synergistic effect of natural compounds combined with chemopreventive agents has been proven and it also serves to mitigate drug-associated toxicities [165]. Epigallocatechin-3-gallate, an active compound of green tea is used for its chemopreventive effects, or to kill cancer cells, and, in combination with chemotherapeutic drugs, it reduces the toxicities and enhances the anti-cancerous activity (Table 3). In this context, a study based on osteosarcoma was performed to evaluate Epigallocatechin-3-gallate and Doxorubicin’s role in the inhibition of osteosarcoma. The result showed that catechin plays a role in the reduction of the doxorubicin-induced pro-survival autophagy [166]. The synergistic approaches were used through combining Epigallocatechin-3-gallate with cisplatin or oxaliplatin to minimize the ill effects of platinum-based therapy. The treatment of colorectal cancer cells with Epigallocatechin-3-gallate and cisplatin or oxaliplatin demonstrated a synergistic effect on inhibition of cell proliferation and induction of cell death. Epigallocatechin-3-gallate enhanced the effect of cisplatin and oxaliplatin-induced autophagy in cancer cells. The finding advocates that cisplatin or oxaliplatin in the presence of EGCG, an active compound of green tea, plays a significant role in the increase the effect of cytotoxicity of cisplatin and oxaliplatin in colorectal cancer cells via autophagy associated pathway [167]. The synergistic effects of Epigallocatechin-3-gallate with sulindac on the inhibition of intestinal tumors in multiple intestinal neoplasia mice was investigated. Treatment with both green tea extract and sulindac significantly decrease the number of tumors, while treatment with green tea extract alone or with sulindac alone reduced it less than combination treatment. The results also showed that green tea extract decreases the tumor growth in Min mice nearly as powerfully as sulindac itself did [168]. Co-treatment with Epigallocatechin-3-gallate and celecoxib powerfully induced the expression of both GADD153 mRNA level and protein cancer cells, although neither Epigallocatechin-3-gallate nor celecoxib alone did. Synergistic effects with the combination were also noticed in lung cancer cell lines. Therefore, upregulation of GADD153 is narrowly correlated with synergistic enhancement of apoptosis with Epigallocatechin-3-gallate and co-treatment also activated the mitogen-activated protein kinase [169]. Epigallocatechin-3-gallate or sulforaphane was used to treat both paclitaxel-sensitive ovarian cancer cell lines alone or in combination. It was reported that sulforaphane inhibits the cell viability of both ovarian cancer cell lines and that Epigallocatechin-3-gallate enhances the inhibiting effect of sulforaphane on ovarian cancer cells. Cell cycle analysis shows that sulforaphane can arrest ovarian cancer cells in G2/M phase, whereas Epigallocatechin-3-gallate and sulforaphane co-treatment can arrest cells in both G2/M and S phase [170]. The low concentrations of catechins are cytotoxic to ER alpha-human breast cancer cells, and the combination of Epigallocatechin-3-gallate and 4-hydroxytamoxifen provokes synergistic cytotoxicity in human breast cancer cells [171]. The effects of the PGHS-2-specific inhibitor celecoxib either alone or in combination with this green tea catechin were studied regarding the expression of interlukin-1-induced tumorigenic factors in human pancreatic adenocarcinoma cells. It was reported that co-incubation of cancer cell with celecoxib and Epigallocatechin-3-gallate synergistically reduced metabolic activity through induction of apoptosis and down-regulated release of pro-angiogenic vascular endothelial growth factor and invasiveness-promoting matrix metalloproteinase-2. Moreover, celecoxib and Epigallocatechin-3-gallate synergistically reduced interlukin-1-induced production of pro-inflammatory interlukin-6 and pro-angiogenic IL-8 [172] (Table 3). 

EGCG and other related molecules or natural compounds resulted in a synergistic anticancer effect that was greater than that of the individual compound alone. Efficacy of epigallocatechin gallate was evaluated via growth of cultured HeLa cells and inhibition of the enzymatic activity of cell surface tea target enzyme, a cancer-associated cell surface-located NADH oxidase. The amount of epigallocatechin gallate needed for prevention according to both criteria was decreased ten times via combination with epicatechin (EC), epigallocatechin (EGC) or (−)-epicatechin-3-gallate (ECG). Such mixtures seem likely to offer cancer protection and therapeutic advantages over those of EGCG alone via depressed toxicity of the mixture to normal cells and for more effective blood delivery of orally-administered catechins to a tumor site [178]. Epicatechin increased apoptosis, the growth inhibition of the human lung cancer cell line, and the inhibition of tumor necrosis factor-alpha released from BALB/c-3T3 cells through epigallocatechin gallate and another tea polyphenol with a galloyl moiety in a dose-dependent way. This finding demonstrated that whole green tea is a more realistic mixture of tea polyphenols for cancer prevention in humans than epigallocatechin gallate alone, and that it is even more effective when it is used in combination with other cancer preventives [179]. A pioneer study was performed to evaluate the potential efficiency of curcumin and epigallocatechin gallate (EGCG) against cancer stem cell and to reveal the molecular mechanisms of their anticancer effects. Finding revealed that curcumin and EGCG combined treatment reduced the cancer stem-like Cluster of differentiation 44-positive cell population. Moreover, curcumin and EGCG specifically inhibited STAT3 phosphorylation and STAT3-NFkB interaction was retained [180].

## 7. Available Concentration and Improvement of Bioavailability of Epigallocatechin Gallate (EGCG)

Green tea and its active compound show a pivotal role in cancer management through modulating various gene activities and inhibiting carcinogenesis steps. In spite of its effectiveness and safety, the role of Epigallocatechin-3-gallate in cancer prevention and therapy is still discussed due to a poor bioavailability. 

Only a small fraction of tea catechins present in the intestinal tract after ingestion tea can be absorbed, and consequently measured to be bioavailable, i.e., present in the blood and tissues or undergoing systemic circulation [181].

The peak plasma concentrations of EGCG, an active compound of green tea, are delivered in 1 to 2 h in healthy subjects with one oral dose in the morning after an overnight fasting period. Such levels reduce slowly to unnoticeable levels after 24 h. The elimination half-life of Epigallocatechin-3-gallate takes place at 3.4 ± 0.3 h [182]. Oral administration of pure EGCG at a dose of 1.6 g in healthy human volunteers [183] produced physiologically relevant plasma EGCG concentrations with the potential to have valuable health effects. While there were variations between individuals, the peak EGCG concentrations were reached between 1.3 and 2.2 h after ingestion and the mean elimination half-life ranged from 1.9 to 4.6 h [184]. Another study based on rats reported that complete bioavailability of EGCG after oral administration was 0.1% [184]. Low bioavailability of green tea compound such as EGCG appears to be linked to its poor membrane permeability and transporter-mediated intestinal efflux [185]. Moreover, blood concentration of EGCG peaked from 1 to 2 h after ingestion, when oral EGCG was absorbed by the intestine [186] and the highest concentration of EGCG in fasting rats and human plasma was 0.156 g/mL and 1.047 g/mL respectively [187]. The study result confirmed that less than five percent of the orally given dose of tea catechins reach systemic circulation [188,189]. It has been designated that the highest plasma concentration of Epigallocatechin-3-gallate was only 0.15 micro mol/L after two cups of green tea were in use in the human body [190]. 

After oral administration of green tea, EGCG is commonly metabolized in a phase 2 conjugation manner through glucuronidation, methylation and sulfation steps in the intestine and liver. Sulfation and Glucuronidation increases the EGCG polarity to increase its solubility and assists their eliminations through urine. Almost ten metabolites have been reported in the form of sulfated, O-methylated and glucuronide conjugates of EGCG, and identified in human plasma [191]. These EGCG glucuronides and sulfates have been commonly found in plasma and urine after green tea consumption [192,193]. Most of these green tea catechins including EGCG are further catabolized by colon microflora, and are eliminated through urine after plasma reabsorption [194]. These metabolites are further catabolized and shortened to C6-C1aromatic and phenolic acids, ultimately excreted out through urine. The results from healthy volunteers consuming green tea have shown elevated hippuric acid (*N*-benzoylglycine) excretion, specifying that EGCG metabolism takes place extensively in the colon [195].

There are numerous factors which limit the role of EGCG pharmacological activities, including oxidative decomposition [196], intestinal pH, poor absorption through intestine, enzymatic conversions to methylation, sulfation, and glucuronic acid metabolites [197,198].

Consequently, several studies have been performed to counter the problems of Epigallocatechin-3-gallate such as poor absorption, rapid metabolism and rapid systemic elimination. 

Several reports prove that nanotechnology-based strategies such as encapsulation, liposome, micelles, nanoparticles and various other formulations can be used as delivery means or to enhance the bioavailability of epigallocatechin-3-gallate are described as.

### 7.1. Liposome/Nanoliposome 

Encapsulation methods including nano emulsions, liposomes, and nanoliposomes are rapidly growing methods, and their application has been proved to enhance the bioavailability of natural compounds. 

The feasibility of using liposomes for intratumor distribution of epigallocatechin-3-gallate and its catechin was investigated. The result demonstrated that nearly no drug molecules were noticed when free Epigallocatechin-3-gallate was given to basal carcinoma cells. Moreover, Epigallocatechin-3-gallate encapsulated in liposomes with deoxycholic acid and ethanol increased drug deposition by twenty times compared to the free form. In addition, liposomes protected Epigallocatechin-3-gallate from degradation, resulting in the induction of greater basal carcinoma cell death compared to that caused by free epigallocatechin-3-gallate at lower concentrations [199]. Epigallocatechin-3-gallate nanoliposome were prepared using ethanol injection method combined with dynamic high-pressure microfluidization. Epigallocatechin-3-gallate nanoliposome showed good physicochemical characterizations and displayed a relatively well sustained release property. Stability of Epigallocatechin-3-gallate in simulated intestinal fluid was considerably improved by nanoliposome encapsulation. After 90 min incubation in SIF without or with pancreatin, the residual of Epigallocatechin-3-gallate nanoliposome was 31.2% and 47.7% respectively, while the residual Epigallocatechin-3-gallate solution was just 3.4% and 3.5% respectively [200]. The anticancer activity of the EGCG-adsorbed pNG was examined and EGCG-pNG was shown to inhibit tumor cell growth by means of cell apoptosis. Moreover, the mechanism of tumor suppression by injecting Epigallocatechin-3-gallate -pNG directly into the tumor site was measured through downregulation of vascular endothelial growth factor. In this measure, the prepared Epigallocatechin-3-gallate -pNG was established to be more powerful than free EGCG in preventing a bladder tumor in model mice [201]. A study based on this result demonstrated that EGCG encapsulating nanoparticles led to an increase anti-proliferative activity in prostate cancer cell lines compared to the free Epigallocatechin-3-gallate. The behavior of this green tea active compound encapsulated in nanoparticles in modulating apoptosis and cell-cycle, was also explained. Then, in vivo experiments, in the mouse xenograft model of a prostatic tumor, using epigallocatechin-3-gallate -loaded NPs, with a model of targeted nanosystems, were conducted. The data supported our hypothesis of target-specific enhanced bioavailability and limited undesirable toxicity. Therefore, it is important to establish the substantial potential in terms of likely clinical outcome [202].

### 7.2. Nanoemulsion

Nanoemulsion based formulations are used to enhance the bioavailability of natural compounds, and encapsulation protects the drug from degradation and increases its half-life in the plasma [203]. Several studies confirmed the role of nanoemulsion in the enhancement of bioavailability of EGCG. A pioneering study was performed to prepare EGCG nanoemulsion (nano-EGCG) to increase the stability and decrease the side effects of EGCG in the treatment of human lung cancer cells. Results confirmed that both EGCG and nano-EGCG inhibited the growth of lung cancer cells, with half-maximal inhibitory concentrations of 36.03 and 4.71 μM, respectively. Moreover, nano-EGCG efficiently repressed lung cancer cell colony formation, migration, and invasion in a dose-dependent manner. Moreover, Nano-EGCG may inhibit lung cancer cell invasion and inhibit lung cancer cell proliferation, colony formation, migration, and invasion [204]. Compared with rats fed an aqueous tea polyphenols solution, rats fed the tea polyphenols nanoemulsion had meaningfully lower maximum plasma concentrations (C_max_) of EGCG and EGC, whereas area under the plasma concentration-time curve (AUC_0-t_) was increased. Moreover, finding show that use of a nanoemulsion system to deliver tea polyphenols may enhance the absorption of EGCG via controlled release [205].

### 7.3. Chitosan/Carbohydrate Based Carrier 

Chitosan has been used as a carrier in polymeric nanoparticles for drug delivery via several routes of administration [206]. A study was performed to synthesize, characterize and assess the efficacy of a nanotechnology-based oral formulation of chitosan nanoparticles encapsulating epigallocatechin-3-gallate (Chit-nanoEGCG) in the treatment of prostate cancer. The antitumor efficacy of Chitnano EGCG was measured in subcutaneously implanted 22Rν1 tumor xenografts in athymic nude mice. Treatment with Chitnano EGCG showed substantial inhibition of tumor growth and secreted prostate-specific antigen levels compared with the EGCG and control groups. Moreover, in the tumor tissues of mice treated with Chit-nano EGCG, compared with groups treated with EGCG and controls, there was a notable induction of poly (ADP-ribose) polymerases cleavage and an increase in the protein expression of Bax with a concomitant decrease in Bcl-2 [207]. The nanoparticle drug carrier system between nanoparticles chitosan and Epigallocatechin-3-*O*-gallate (EGCG) was made to inhibit breast cancer cells. Findings revealed that the chitosan-EGCG nanoparticles showed an inhibitory effect on the growth of breast cancer cells. The inhibition rate could reach up to 21.91% [208]. (+)-catechin (C) and (−)-epigallocatechin gallate (EGCG) were encapsulated in chitosan nanoparticles (CS NP) as a means of improving their intestinal absorption. Results demonstrated that cumulative amounts transported after encapsulation were significantly higher for C and EGCg, respectively. This study establishes that encapsulation of catechins in chitosan nanoparticles improves their intestinal absorption and is an encouraging approach for improving their bioavailability [209].

### 7.4. Polymeric Nanoparticles 

Polymeric nanoparticles are widely engaged as biomaterials because of their encouraging characteristics in terms of simple amplification and design, better biocompatibility, a wide-ranging structures diversity and clear bio-imitative characteristics [210]. pH-sensitive polymeric nanoparticles of EGCG were prepared and evaluated. The therapeutic efficacy of EGCG NPs on chronic kidney disease was examined on models of rat Nephrotic syndrome. EGCG NPs could meaningfully modify the pharmacokinetic profile and increase the bioavailability of EGCG by more than 2.4-times in comparison with the EGCG powder group. At the end of the fourth and sixth week, proteinuria excretion of nephrotic syndrome rats treated with Epigallocatechin gallate NPs was significantly lower than those treated with EGCG powder [211]. Polymeric EGCG-encapsulated nanoparticles (NPs) were prepared and targeted with small molecular entities capable of binding to prostate specific membrane antigen, a transmembrane protein that is overexpressed in prostate cancer, and their efficacy was assessed in preclinical studies. The resultant Epigallocatechin gallate encapsulating nanoparticles led to an improved anti-proliferative activity in prostate cancer cell lines compared to the free Epigallocatechin gallate [202]. 

### 7.5. Serum Albumin and Caseins Used as a Carrier 

Various proteins including bovine serum albumin and casein have been broadly used in drug-delivery based research. The ability of casein micelles to deliver biologically active concentrations of polyphenols to colon cancer cells was tested. The cytotoxicity and proliferation behavior of colon cancer cells when exposed to free EGCG was compared with that of nanoencapsulated EGCG in casein micelles of skim milk. Results revealed that EGCG-casein complexes were capable of decreasing the proliferation of cancer cells, representing that bioavailability may not be reduced by the nanoencapsulation. Casein micelles may act as protective carriers for EGCG in foods [212]. A pioneering study was designed to increase the tea polyphenols’ bioavailability by nanoformulation by using bovine serum albumin as the matrix and chitosan as the external shell. Encapsulated tea polyphenols nanoparticles were spherical in size and promoted tea polyphenols’ stability in normal and gastrointestinal conditions. Encapsulated tea polyphenols have shown a meaningfully higher level of radioprotection than tea polyphenols, proposing that tea polyphenols nanoparticles can be discovered as a valued radioprotective and pharmacotherapeutic agent [213].

### 7.6. Structural Modification of EGCG

The compound (−)-epigallocatechin-3-*O*-(3-*O*-methyl) gallate (EGCG3′′Me), an *O*-methylated derivative of EGCG, was made and tested. It was determined that EGCG3′′Me has a significant inhibitory effect on the activity of angiotensin I-converting enzyme (ACE). The effect of Benifuuki tea on human hypertension is chiefly the result of the strong inhibitory effect of EGCG3′′Me on angiotensin I-converting enzyme activity, its high rate of absorption, and its stability in the blood [214]. Epigallocatechin-3-*O*-gallate acetylated derivatives were made using lipase catalyzed acylation of EGCG with vinyl acetate to improve its lipophilicity. The immobilized lipase, Lipozyme RM IM, was found to be the optimum catalyst. The antioxidant activity of the acetylated EGCG derivatives were superior to butylated hydroxytoluene (BHT), tert-butyl hydroquinone (TBHQ) and EGCG. Acetylated EGCG showed the highest 1,1-Diphenyl-2-picrylhydrazyl radical scavenging activity compared to EGCG, BHT and TBHQ [215]. 

## 8. Conclusions

Despite considerable recent progress, cancer continues to represent a major cause of mortality and morbidity worldwide. It is a notorious killer, and risk factors linked with cancer seem to be increasing day by day. Anticancer drugs are effective in the treatment of cancer but cause adverse side effects including fatigue, hair loss, infection, nausea and vomiting, appetite complications and changes in physiological and biochemical processes. Natural products have been shown to play significant role in cancer prevention and inhibition through modulating various biological activities. Epigallocatechin-3- gallate, the most abundant catechin in tea, and its implication in health care and disease prevention has been described. EGCG is reported to possess antioxidant, anti-inflammatory and anticancer activities. Preclinical and clinical evidence clearly shows that EGCG plays a significant role in the inhibition and prevention of cancer. Cancer development and progression is a multi-step process and normal cells convert to the metastatic stage through carcinogenesis. EGCG shows an anti-cancerous effect via inhibition of initiation, promotion and progression stages. The additive or synergistic effect of EGCG with chemopreventive agents has been proven to enhance the anti-cancerous activity and reduce the toxicities. Poor bioavailability, rapid metabolism and fast elimination of EGCG compound caused a limitation of this compound in health management. However, nanotechnology-based strategies are being used as delivery means to enhance the bioavailability of EGCG.

## Figures and Tables

**Table 2:** Role of EGCG in cancer prevention through based on in vitro study.

Cancer Types	Study Type	Finding of the Study	Refs.
Cervix cancer	In vitro	EGCG with eugenol amrogentin greatly inhibit the cellular proliferation and colony formation	[44]
Cervix cancer	In vitro	EGCG treatment causes down regulation of genes involved in the stimulation of proliferation and motility and invasion processes.	[46]
Breast cancer	In vitro	EGCG reduced breast cancer cell growth in a concentration- and time dependent manner	[48]
Breast cancer	In vitro	Epigallocatechin gallate powerfully inhibited the growth of cancer stem/progenitor cells.	[49]
Breast cancer	In vitro	Protein expression of HIF-1α and VEGF dropped in cancer cells pre-treated with increasing concentrations of	[51]
Ovarian cancer	In vitro	EGCG improved the toxicity of cisplatin and epigallocatechin-3-gallate increased cisplatin strength	[52]
Ovarian cancer	In vitro	EGCG plays an important role in decreasing ovarian cancer cell growth. Correspondingly, Epigallocatechin gallate showed growth inhibitory effects in each cell line in a dose-dependent approach and induced apoptosis and cell cycle arrest	[56]
Ovarian cancer	In vitro	Epigallocatechin-3-gallate causes a substantial task in decreasing cancer cell growth, showed dose dependent growth inhibitory effects	[57]
Endometrial cancer	In vitro	EGCG caused the arrest of cells in the G0/G1 phase of the cell cycle	[62]
Endometrial cancer	In vitro	EGCG was established to inhibit proliferation of adenocarcinoma cells	[63]
Pancreatic cancer	In vitro	EGCG decreased pancreatic cancer cell migration, growth and invasion	[67]
Pancreatic cancer	In vitro	EGCG reduced pancreatic cancer cell growth in a concentration-dependent manner	[68]
Pancreatic cancer	In vitro	The synergistic activity was credited to the cell cycle arrest and the induction of the reactive oxygen species-dependent mitochondria mediated apoptosis	[70]
Pancreatic cancer	In vitro	EGCG caused growth arrest at G1 stage of cell cycle, and induced apoptosis	[72]
Gastric cancer	In vitro	EGCG was accomplished to inhibit vascular endothelial growth factor secretion and expression	[74]
Gastric cancer	In vitro	EGCG significantly inhibited proliferation and increased apoptosis of cancer cells in vitro.	[75]
Gastric cancer	In vitro	EGCG meaningfully promoted apoptosis and inhibited the proliferation	[77]
Gastric cancer	In vitro	EGCG treatment reduced vascular endothelial growth factor protein level	[78]
Gastric cancer	In vitro	Microvessel density in tumor tissues receiving epigallocatechin-3-gallate treatment was also evidently reduced and markedly reduced VEGF protein level	[79]
Liver tumour	In vitro	The epigallocatechin gallate reduced hypoxia-incited apoptosis in HepG2 cells as well as enhanced cell survival	[82]
Liver cancer	In vitro	Epigallocatechin gallate reduced expression of MMP-9, syndecan-1 and FGF-2	[83]
Colorectal cancer	In vitro	Epigallocatechin gallate and sodium butyrate combination treatment induced apoptosis and cell cycle arrest	[85]
Colon cancer	In vitro	EGCG-induced downregulation of epidermal growth factor receptor cancer cells	[86]
Colon cancer	In vitro	Both Epigallocatechin-3-gallate and Poly E initiated a decrease in the phosphorylated forms of EGFR	[87]
Bile duct cancer	In vitro	JAK/STAT pathway activation through pro-inflammatory cytokine in cancer cells was decreased via pre-treatment with quercetin and epigallocatechin-3-gallate	[91]
Bile duct cancer	In vitro	The combination of vorinostat and epigallocatechin-3-gallate revealed synergistic growth inhibitory effects and caused induction of apoptosis in tumor cells.	[92]
Renal Cell Carcinoma	In vitro	Epigallocatechin-3-gallate inhibits growth and induces apoptosis	[94]
Renal Cell Carcinoma	In vitro	EGCG showed potentiality to inhibit the proliferation, and induce apoptosis	[95]
Renal Cell Carcinoma	In vitro	EGCG treatment provoked important upregulation of Cx32 in cancer cells	[97]
Prostate Cancer	In vitro	EGCG induces apoptosis through triggering caspase and preventing the expression of Bcl-2	[99]
Prostate Cancer	In vitro	Epigallocatechin-3-gallate demonstrated low inhibitory effect on cancer cell proliferation	[100]
Prostate Cancer	In vitro	EGCG showed anticancer effects and it was proved that epigallocatechin-3-gallate inhibited cancer cell proliferation	[102]
Urinary bladder cancer	In vitro	Treatment of EGCG caused in important inhibition of cell proliferation via induction of apoptosis and inhibited cancer cell migration	[104]
Urinary bladder cancer	In vitro	Epigallocatechin-3-gallate increased growth inhibition in a dose- and time-dependent manner	[105]
Leukemia	In vitro	Proliferation and cell cycle progression of cancer cells treated with epigallocatechin-3-gallate were inhibited	[109]
Leukemia	In vitro	Epigallocatechin-3-gallate treatment induced apoptosis and increased the levels of Bax protein expression	[111]
Leukemia	In vitro	EGCG showed higher growth suppression and induced apoptosis demonstrated by nuclei fragmentation and nuclear fragmentation	[113]
Lymphoma	In vitro	EGCG induced growth inhibition and apoptosis in a dose- and time-dependent way	[114]
Lymphoma	In vitro	Epigallocatechin-3-gallate were able to inhibit the growth of malignancy cell lines	[115]
Lymphoma	In vitro	EGCG caused induction of cell death and reactive oxygen species generation	[116]
Head and neck cancer	In vitro	EGCG inhibits the self-renewal capacity and reduces the expression of stem cell markers	[118]
Head and neck cancer	In vitro	EGCG induces apoptosis of cancer cells via regulating Bim and Bcl-2	[119]
Head and neck cancer	In vitro	Combined treatment with erlotinib and EGCG inhibited the protein level of p65 subunit of nuclear factor-kappaB	[120]
Oral cancer	In vitro	EGCG inhibited cell viability in a time- and concentration-dependent manner	[122]
Oral cancer	In vitro	Epigallocatechin-3-gallate in inhibiting HGF-induced tumor growth and invasion	[124]
Oral cancer	In vitro	EGCG caused an inhibitory effect on cell migration, motility, spread, and adhesion	[125]
Oesophagus cancer	In vitro	Epigallocatechin-3-gallate considerably reduced the invasion and viability capacity of cancer cells	[127]
Oesophagus cancer	In vitro	Epigallocatechin-3-gallate inhibited proliferation of cancer cells	[128]
Lymphoma	In vitro	Vorinostat alone or in combination with epigallocatechin-3-gallate imparts anti-proliferative effects	[130]
Lymphoma	In vitro	EGCG-induced inhibition of tumor cell proliferation	[132]
Lung cancer	In vitro	EGCG decrease the expression of both Axl and Tyro 3 receptor tyrosine kinases	[139]
Myeloma	In vitro	The treatment of the cancer cell line with epigallocatechin-3-gallate inhibits cell proliferation as well induces apoptosis	[141]
Myeloma	In vitro	EGCG inhibited the effect of endothelial cell migration induced and the numbers of migrated cells and numbers of migrated cells	[142]
Osteosarcoma	In vitro	EGCG has an anticancer effect on cancer cells	[144]
Osteosarcoma	In vitro	EGCG showed role in the suppression of proliferation of cancer cells in a concentration-dependent and time-dependent manner	[145]
Brain tumor	In vitro	EGCG induced apoptosis in glioma cells.	[147]
Brain tumor	In vitro	EGCG treatment leads to a decrease in cell viability and the S-phase cell fraction	[149]
Thyroid cancer	In vitro	EGCG decreased the migration and invasion,	[151]
Thyroid cancer	In vitro	EGCG considerably suppresses invasion and migration in anaplastic cancer cells	[152]
Retinoblastoma	In vitro	EGCG treatment of cancer cells resulted in a dose- and time-dependent decrease in the total pRb	[154]

**Table 3:** Synergistic effects of combination of EGCG and other anticancer drugs.

EGCG + Anticancer Compound	Type of Cancer	Outcome of the Study	Refs.
**EGCG + Doxorubicin**	Osteosarcoma	Epigallocatechin-3-gallate reduce the Doxorubicin-induced pro-survival autophagy	[166]
**EGCG + Cisplatin or oxaliplatin**	Colorectal cancer	Treatment of colorectal cancer cells with Epigallocatechin-3-gallate and cisplatin or oxaliplatin confirmed a synergistic effect on inhibition of cell proliferation and induction of cell death.	[167]
**EGCG + Sulindac**	Intestinal nepoplasia	Treatment with both green tea extract and sulindac significantly decrease the number of per mouse	[168]
**EGCG + Celecoxib**	Prostate cancer	Co-treatment with epigallocatechin-3-gallate and celecoxib powerfully induced the expression of both GADD153 mRNA level and protein	[169]
**EGCG + Sulforaphane**	Ovarian cancer	Sulforaphane inhibits cell viability of cancer cell and epigallocatechin-3-gallate enhance the inhibiting effect of sulforaphane	[170]
**EGCG + Hydroxytamoxifen**	Breast cancer	The combination of EGCG and 4-hydroxytamoxifen provokes synergistic cytotoxicity in cancer	[171]
**EGCG + Celecoxib**	Pancreatic cancer	Co-incubation of cancer cells with celecoxib and epigallocatechin-3-gallate synergistically reduced metabolic activity through induction of apoptosis	[172]
**EGCG + SU5416**	Neuroblastoma	Combination of drugs can be a promising therapeutic strategy for controlling the growth of neuroblastoma cells.	[173]
**EGCG + Sulforaphane**	Colon cancer	Low and high dose combinations of Sulforaphane and epigallocatechin-3-gallate attenuated the cellular senescence induced by epigallocatechin-3-gallate alone	[174]
**EGCG + Tamoxifen**	Breast cancer	Tamoxifen at realistic dose suppress the growth of ER-negative breast cancer when combined with Epigallocatechin-3-gallate.	[175]
**EGCG + Taxane**	Prostate cancer	Epigallocatechin-3-gallate in combination with taxane may provide a novel therapeutic treatment of prostate cancer	[176]
**EGCG + Doxorubicin**	Prostate cancer	Epigallocatechin-3-gallate combined with Doxorubicin may have significant clinical application in the treatment of metastatic prostate cancer	[177]
